# Screening of cannabis-related problems among youth: the CPQ-A-S and CAST questionnaires

**DOI:** 10.1186/1747-597X-7-13

**Published:** 2012-04-02

**Authors:** Sergio Fernandez-Artamendi, José Ramón Fernández-Hermida, José Muñiz-Fernández, Roberto Secades-Villa, Gloria García-Fernández

**Affiliations:** 1 Addictive Behaviours Research Group, Department of Psychology, University of Oviedo, UCA, Facultad de Psicología, Pza Feijoo s/n, 33003 Oviedo (Asturias), Spain; 2 Addictive Behaviours Research Group, Department of Psychology, University of Oviedo, Office 205, Facultad de Psicología, Pza Feijoo s/n, 33003 Oviedo (Asturias), Spain; 3 Addictive Behaviours Research Group, Department of Psychology, University of Oviedo, Office 217, Facultad de Psicología, Pza Feijoo s/n, 33003 Oviedo (Asturias), Spain; 4 Addictive Behaviours Research Group. Department of Psychology, University of Oviedo, Office 201, Facultad de Psicología, Pza Feijoo s/n, 33003 Oviedo (Asturias), Spain; 5 Addictive Behaviours Research Group, Department of Psychology, University of Oviedo, UCA, Pza Feijoo s/n, 33003 Oviedo (Asturias), Spain

**Keywords:** CAST, CPQ-A-S, Test, Psychometrics, Cannabis, Adolescents, Young people, Screening, Early detection

## Abstract

**Background:**

Cannabis use among young people is a significant problem, making particularly necessary validated screening instruments that permit secondary prevention. The purpose of this study was to analyze and compare the psychometric properties of the CAST and CPQ-A-S questionnaires, two screening instruments specifically addressing the youth population.

**Methods:**

Information was obtained on sociodemographics, frequency of substance use, psychopathological symptoms and cannabis-use problems, and the CPQ-A-S and CAST were applied, as well as an infrequency scale for discarding responses made randomly. The sample was made up of 144 young people aged 16 to 20 that had used cannabis in the last month, of which 71.5% were boys. Mean age of the sample was 17.38 years (SD = 1.16).

**Results:**

The results show that from the psychometric point of view both the CAST and the CPQ-A-S are good screening instruments.

**Conclusions:**

The CAST is shorter and presents slightly better internal consistency than the CPQ-A-S. Both instruments show high sensitivity and specificity in the detection of young people dependent on cannabis according to the DSM IV-TR criteria. The CPQ-A-S appears to show greater capacity for detecting psychopathological distress associated with use. Both questionnaires yield significant odds ratios as predictors of frequent cannabis use and of the DSM IV-TR abuse and dependence criteria. In general, the CPQ-A-S emerges as a better predictor than the CAST.

## Background

Prevalence of cannabis use among adolescents is very high. In Europe the most recent data [1]on prevalence of use in those aged 15 and 16, according to the *European Monitoring Centre for Drugs and Drug Addiction*, indicate lifetime prevalence of between 4% and 45%, annual prevalence of 2% to 35% and monthly prevalence of 1% to 20%, depending on the country. In the most highly populated countries (Germany, France, United Kingdom, Spain, Italy), more than 20% report having used cannabis at some time in their life, more than 15% in the last year, and over 7% in the last month [1]. As in most European countries, figures have stabilized in Spain since the beginning of the 2000s [2]. However, Spanish prevalence of cannabis use in lifetime (37%), last year (30%) and last month (20%) is among the highest [1]. Furthermore, age at onset of cannabis use among Spanish adolescents has decreased from 15.1 years in 1994 to 14.6 in 2008 [3].

This widespread use has been accompanied by a growing awareness of the dangers of cannabis. Numerous studies have linked it to cognitive impairment [4] and to increased vulnerability to suffering from certain psychopathological disorders (or to their worsening) [5] that already typically affect adolescents [6]. This perception of danger does not appear to have made much impression on young consumers, who do not perceive the use of this drug as problem, and tend not to request help [7]. It is common for drug users not to seek professional help until they have a long history of use of the drug [8]. This is of great concern, since even occasional use has been related to later drug use and educational problems [9]. This situation makes it essential to use reliable and valid screening methods [10,11] that permit early detection of at-risk users [12], so that they can be referred to early intervention programmes.

Currently, there are very few brief screening instruments aimed specifically at the young population, and those that do exist are in need of further validation in different cultures and populations [13]. Among the few available is the *Cannabis Abuse Screening Test *(CAST) [14], a 6-item instrument designed specifically to detect patterns of cannabis abuse in adolescents and young people, and which focuses on difficulties for controlling use and on the negative consequences for health or social relations. This questionnaire was recently included in the European School Survey Project on Alcohol and Other Drugs, the ESPAD [15].

Another available instrument is the CPQ-A-S [11], a shortened, 12-item version of the *Cannabis Problems Questionnaire for Adolescents *[16], whose full, 27-item version was designed as an aid to the assessment and diagnosis process. According to the authors [11], this briefer version is conceived as a useful tool for the detection of young people at high risk for cannabis use, in conditions that are unfavourable to the use of more extensive instruments. Its utility was partially shown in the original study [11], where scores on the scale emerged as associated with intensity of cannabis use.

Instruments for early detection should be easily and quickly applied, affordable, and reasonably accurate [[17], p. 222]. Consequently, screening instruments, in addition to being brief and simple to administer, should have high predictive validity and high sensitivity and specificity in the detection of possible risk cases. Both the CPQ-A-S and the CAST meet the criteria for screening instruments.

However, there are no studies that have analyzed the differences between these two tools in relation to their capacity as instruments for the early detection of young people at high risk for cannabis use. The aims of the present research are (1) to analyze the structure, reliability and validity of the CAST and CPQ-A-S, (2) to determine their sensitivity and specificity to detect cannabis dependence, and (3) to compare their capacity to detect other cannabis-related problems.

## Method

### Participants

The initial sample was made up of 1069 students from high school and technical/vocational courses (*Bachillerato *and *Formación Profesional*) at 9 schools in the Principality of Asturias, a region in the north of Spain. The schools were randomly selected from those areas of the region with the highest prevalence of cannabis use, so as to guarantee the maximum numbers of users. Participation in the study was voluntary and none of the students refused to participate. The returned questionnaires were screened by means of an Infrequency Scale which detected responses made erroneously or at random. Among participants delivering invalid surveys there were significantly (*p *< .05) more students of foreign origin, which presumably indicates language difficulties. After the removal of 191 invalid questionnaires with such responses (17.87% of the initial sample), a total of 878 valid questionnaires were obtained. Of these, 130 were discarded because they had been filled out by participants outside the age range of the study. The resulting sample was made up of 748 students aged 16 to 20 years (M = 17.12, SD 1.17). The final study sample was confined to those participants who had used cannabis in the past month, and who could therefore complete the CAST and CPQ-A-S together with the rest of the instruments. A total of 144 students filled out both instruments.

### Procedure

The CPQ-A-S and the CAST, as described above, were applied together with a battery of tests by means of a computerized procedure developed with the Lime Survey^® ^software. The program presented to participants only those items applicable to their personal situation according to the information previously provided. Moreover, the software was set up to alert respondents if they gave an incongruent answer and to prevent them returning the questionnaire unless all the questions were answered. Ethical approval for the research was obtained, as well as permissions from both the schools and the Education Department of the Principality of Asturias. Informed consent for all participants was obtained through educational institutions. No identifying information was requested, and confidentiality of responses was guaranteed.

Participants filled out the questionnaires during school time, in a classroom with Internet access and in a single session, where no teaching staff were present. A researcher supervised the session, guaranteeing that students respected the privacy of their partners, answering any questions they may have about the survey, and staying away from student's computers. The survey presented participants with questions on the following topics, in this order.

### Measures

#### Sociodemographic information

Participants were required to provide information on their age, sex and place of birth (national/foreigner).

#### Substance use

Frequency and patterns of substance use were assessed by means of items from the European School Survey Project on Alcohol and Other Drugs Student Questionnaire 2007 (ESPAD) [18]. The questions were designed to obtain information on the prevalence of cannabis use, but also the use of tobacco, alcohol and other illegal drugs during the last week, the last month, the last year and throughout one's whole life up to that point. Response options for each time period were: never, 1-2 times, 3-5 times, 6-9 times, 10-19 times, 20-39 times and 40 times or more. Intensive cannabis use was defined as using it 10 times or more in the previous month.

#### Assessment of problems due to cannabis use

Presence of cannabis abuse and dependence was assessed by means of two sets of self-reported questions [7] on the presence of the corresponding DSM-IV-TR [19] criteria for these diagnoses in the previous 12 months. The presence of a diagnosis of cannabis dependence is a significant threshold for problematic cannabis use, distinguishing experimental use from a high-risk situation for future problems. Therefore, problematic use is defined in this study as presenting at least three symptoms of dependence, as assessed by the DSM-IV-TR.

Level of general concern deriving from cannabis use was rated by means of a Likert-type scale ranging from 0 (no concern) to 10 (very high concern). Furthermore, specific areas of concern were explored through dichotomous questions on various areas of the cannabis user's life that might be affected (academic performance, family relations, relations with friends, intimate partner relations, memory, health, future, use of other drugs, ability to have fun without drugs). It is considered that higher score in degree of concern and a larger number of areas affected will be associated with higher score on the screening instruments.

Both CPQ-A-S and CAST were applied in Spanish. The CPQ-A-S was obtained from the Spanish version of CPQ-A [20], and content equivalence with the original version was guaranteed by following the guidelines of the International Test Commission [21]. The CPQ-A-S used a dichotomous response option (yes/no), as stipulated by the authors [11]. Regarding the CAST [14,22], we utilized the full Spanish version [23], which consists of five response options (from 1: never, to 5: very often).

#### Psychopathological symptoms

Psychopathological problems were assessed by means of the Brief Symptom Inventory (BSI) [24]. This instrument yields scores on the following 9 dimensions: somatization, obsessive-compulsive, interpersonal sensitivity, depression, anxiety, hostility, phobic anxiety, paranoid ideation and psychoticism. Reliability of each one of the BSI dimensions in Spanish samples ranges from 0.72 to 0.95 [25].

#### Problems due to alcohol use

For the assessment of problems deriving from drinking we used the Spanish version [26] of the Rutgers Alcohol Problems Index (RAPI) [27]. This questionnaire is made up of 23 items with Likert-type response on the frequency of various consequences of excessive alcohol consumption.

#### Infrequency scale

An infrequency scale was included in the survey with the aim of detecting those questionnaires that had been responded to in a random or erratic manner. The instrument selected was the Oviedo Infrequency Scale [28], comprising 12 Likert-type items with five response options on the respondent's degree of agreement or disagreement with items of the type "I know people that wear glasses". Items were randomly distributed throughout the questionnaire. Those surveys with more than three erroneous responses were discarded from subsequent analyses, in accordance with the instructions of the scale's authors [28].

### Data analysis

Exploratory factor analyses were carried out to determine the dimensional structure of the instruments. The fit indices used for the factorial model are the RMSEA (Root Mean Square Error of Approximation) and CFI (Comparative Fit Index). Diagonals were estimated with Weighted Least Square Mean-adjusted parameter (WLSM), which uses a diagonal weight matrix with standard errors and mean-adjusted chi-square.

We then calculated internal consistency using the Cronbach's alpha coefficient for both instruments, before obtaining the discrimination index of the items of both scales by means of the corrected item-total correlation.

The Pearson correlation between total scores on the questionnaires was obtained in order to determine the degree of convergence between them. We also used the canonical correlation [29,30] to estimate the degree of convergence between the items of the two instruments.

To analyze the predictive validity we calculated the ROC curve, with a view to determining the optimum cut-off point for both instruments to predict the presence of cannabis dependence, following criteria from previous psychometric studies [22]. Areas obtained under ROC Curves were compared. Sensitivity, specificity and percentage of participants correctly classified by the cut-off point were calculated, with a Confidence Interval of 95%.

Next, we used bivariate analyses and their effect sizes to check the discriminative capacity of the resulting cut-off points of the CAST and CPQ-A-S for detecting significant differences in additional indicators of cannabis-related problems: concern about the effects of use, presence of psychopathological symptoms, alcohol consumption, use of tobacco and other illegal drugs, and problems deriving from alcohol use (RAPI). In the case of continuous variables we calculated the Student t and Cohen's d for estimating effect size. In the case of categorical variables we used the Chi-squared statistic and Cramer's V to estimate effect size. This allowed us to obtain a profile of problematic cannabis user detected by the optimum cut-off points

Finally, we carried out logistic regressions on intensive use of cannabis in the past month, any cannabis use in the past week, and presence of cannabis dependence or abuse according to the DSM-IV-TR criteria.

For the analyses we used the statistics packages Mplus 5.2 (factor analyses) and SPSS for Windows, version 15, except in the case of Cohen's d, which was calculated through application of the corresponding formula.

## Results

### Sociodemographic characteristics and drug use

A total of 144 participants reported any cannabis use in the month prior to the survey, therefore filling out the CPQ-A-S and CAST. Mean age of the sample was 17.38 years (SD = 1.16), including 71.5% boys. The majority of them (97.9%) were living with their families, whereas 2.1% lived in residential care. Regarding their school level, 46.9% of them were students from technical/vocational courses (*ciclos formativos*) and 53.1% were in high school (*Bachillerato*). A total of 84.2% reported any tobacco use in the previous month, 93.1% had drunk alcohol and 64.1% had got drunk. Number of respondents using cannabis at least weekly in the previous month was 54.5%. In the week prior to the survey, 42.1% did not smoke cannabis and 22.5% used it on a daily basis. Of this final sample, 14.5% were cannabis abusers and 31.9% were cannabis-dependent, according to the DSM IV-TR.

### Factor analysis

The exploratory factor analysis of the CPQ-A-S yielded an RMSEA of 0.041 and a CFI of 0.964 for a one-factor solution. Therefore, the questionnaire items show a satisfactory fit to a one-dimensional model, with an eigenvalue of 4.373. Factor weights of the items are shown in Table 1 where it can be observed that the highest weights correspond to items 11, 9 and 5, respectively.

**Table 1:** Factor weights of the CPQ-A-S items for a single-factor solution

Items	Factor weights
1 Smoke more on your own	.548

2 Worried meeting people when stoned	.336

3 More time with smoking friends	.522

4 Criticized for smoking too much	.558

5 Worried money spent on cannabis	.754

6 Trouble with police	.517

7 Physically sick after smoking	.458

8 Passed out after smoking	.377

9 Pains inchest or lungs after smoking	.774

10 Persistent chest infection/cough	.520

11 Paranoid or antisocial after smoking	.820

12 Worried losing friends or family	.498

The result of the exploratory factor analysis of the CAST provides an RMSEA of 0.208 and a CFI of 0.972 for a one-factor solution, with an eigenvalue of 4.089. Even though in the case of a two-factor solution a better fit is obtained (RMSA = 0.00; CFI = 1.00), the two factors are highly correlated (0.649), giving grounds for the assertion that the instrument has an essentially one-dimensional structure. Factor weights of the items are shown in Table 2.

**Table 2:** Factor weights of the CAST items for a single-factor solution

Items	Factor weights
1 Cannabis before midday	.929

2 Cannabis when alone	.913

3 Memory problems	.814

4 Friends or family	.818

5 Tried to reduce/to stop	.658

6 Problems	.697

### Reliability

Cronbach's alpha coefficient of the CPQ-A-S is 0.70, and of the CAST, 0.84. Correlation between the total scores of the two scales is 0.58, and the associated variance is 33.64%.

Discrimination indexes of the CAST items are high, with values of over 0.40 for all of them (.710, .707, .699, .596, .445, and .579). This confirms once more the essential one-dimensionality of the scale. Discrimination indices of the CPQ-A-S are lower than those of the CAST, with four items with values below 0.30.

### Validity

Correlations between the items of the two scales are reasonably high (Table 3), so that the canonical correlation between the two blocks of items taken together is 0.663. This indicates a high degree of convergence between the two instruments, with 44% of associated variance. The items that contribute most to this correlation are 1, 8 and 10 of the CPQ-A-S and 2 and 3 of the CAST.

**Table 3:** Pearson correlations between items of the CAST and the CPQ-A-S

		**CAST Items**					
		
		**1**	**2**	**3**	**4**	**5**	**6**
	
**CPQ Items**	**1**	.44	.52	.25	.11	.14	.14
	
	**2**	.20	.21	.19	.08	.07	.08
	
	**3**	.27	.24	.20	.09	.16	.09
	
	**4**	.12	.17	.22	.49	.06	.21
	
	**5**	.33	.32	.48	.39	.24	.23
	
	**6**	.21	.31	.27	.21	.19	.28
	
	**7**	.16	.18	.17	.14	.02	.11
	
	**8**	.11	.13	.20	.24	.12	.18
	
	**9**	.21	.20	.36	.32	.14	.10
	
	**10**	.28	.29	.25	.15	.22	.19
	
	**11**	.28	.27	.37	.27	.14	.18
	
	**12**	.07	.06	.17	.26	.14	.09

**Figure 1:**
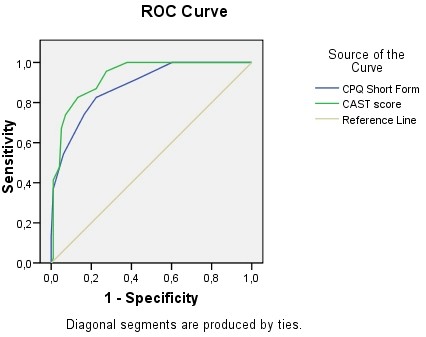
**ROC curves for the CAST and CPQ-A-S instruments**. The figure shows the ROC curve with Specificity and Sensitivity of the Cannabis Abuse Screening Test (CAST) and the Cannabis Problems Questionnaire for Adolescents - Short Form (CPQ-A-S) to detect Cannabis Dependence, as assessed by DSM-IV-TR.

The optimum cut-off point according to the ROC curve (see Figure 1) for detecting the presence of cannabis dependence following DSM IV-TR criteria is 3 points on the CPQ-A-S. This threshold shows sensitivity of 83% (CI95%: 68.6-92.2) and specificity of 77.5% (CI95%: 68.0-85.4). Optimum cut-off point to maximize detection of cannabis dependence is 5 on the CAST, with sensitivity of 83% (CI95%: 58.9-85.7) and specificity of 87% (CI95%: 85.8-97.1). Percentage of correctly classified participants using these cut-off points was 79.2% (CPQ-A-S) and 85.4% (CAST).

Areas under ROC curve were .881 (CI95%: .825-.937) for the CPQ-A-S and .929 (CI95%: .888-.969) for the CAST. Comparison between areas under ROC Curves of CPQ-A-S and CAST showed no statistically significant differences (*p *= .122)

Bivariate analyses were carried out to determine the discriminative capacity of cut-off points for detecting additional cannabis-related problems: severity of cannabis use patterns, psychopathological symptoms and other substances use (Table 4). Scores above cut-off points of CPQ-A-S and CAST indicate significantly earlier age of onset, more years of use, greater degree of concern and greater number of areas of concern (*p *< .05). Participants above CPQ-A-S threshold present significantly (*p *> .05) more severe symptomatology in all 13 subscales of BSI; and in 5 subscales in the case of CAST.

**Table 4:** Concurrent predictive validity of the CPQ-A-S and the CAST using cut-off points to maximize detection of cannabis dependence

Continuous variables	CPQ-A-S					CAST				
	
	Cut-off point	Mean	t	Sig (2-tailed)	Cohen's d	Cut-off point	Mean	t	Sig (2-tailed)	Cohen's d
Cannabis use patterns										

Age at first cannabis use	- 3	15.6	4.206	≤ .001	.71	- 5	15.6	4.166	≤ .001	.73
								
	≥ 3	14.6				≥ 5	14.6			

Years since first cannabis use	- 3	1.78	-3.020	.003	.51	- 5	1.80	-3.247	.001	.57
								
	≥ 3	2.58				≥ 5	2.68			

Global degree of concern about effects of cannabis	- 3	2.36	-4.937	≤ .001	.83	- 5	2.76	-3.398	.001	.56
								
	≥ 3	4.77				≥ 5	4.45			

Number of areas object of concern	- 3	1.96	-3.591	≤ .001	.61	- 5	2.16	-3.062	.003	.53
								
	≥ 3	3.90				≥ 5	3.88			

Psychopathological symptoms										

BSI - Somatization symptoms	- 3	.219	-4.161	≤ .001	.77	- 5	.295	-1.902	.059	
								
	≥ 3	.516				≥ 5	.431			

BSI - Obsessive compulsive symptoms	- 3	.271	-3.525	.001	.64	- 5	.336	-2.167	.032	.38
								
	≥ 3	.597				≥ 5	.534			

BSI - Interpersonal sensitivity symptoms	- 3	.276	-3.628	≤ .001	.67	- 5	.373	-1.851	.066	
								
	≥ 3	.684				≥ 5	.578			

BSI - Depressive symptoms	- 3	.437	-4.458	≤ .001	.81	- 5	.543	-2.297	.023	.40
								
	≥ 3	.891				≥ 5	.779			

BSI - Anxiety symptoms	- 3	.287	-3.966	≤ .001	.72	- 5	.371	-1.841	.068	
								
	≥ 3	.622				≥ 5	.528			

BSI - Hostility symptoms	- 3	.324	-4.271	≤ .001	.80	- 5	.437	-2.170	.032	.38
								
	≥ 3	.795				≥ 5	.672			

BSI - Phobic symptoms	- 3	.291	-2.869	.005	.48	- 5	.358	-.905	.367	
								
	≥ 3	.515				≥ 5	.432			

BSI - Paranoid symptoms	- 3	.274	-3.476	.001	.63	- 5	.368	-1.353	.178	
								
	≥ 3	.612				≥ 5	.499			

BSI - Psychoticism symptoms	- 3	.257	-4.086	≤ .001	.76	- 5	.371	-1.668	.098	
								
	≥ 3	.672				≥ 5	.538			

BSI - Additional symptoms	- 3	.125	-2.827	.006	.53	- 5	.188	-1.083	.281	
								
	≥ 3	.346				≥ 5	.270			

BSI - Global severity index	- 3	.280	-4.362	≤ .001	.81	- 5	.368	-2.051	.042	.36
								
	≥ 3	.630				≥ 5	.532			

BSI - Total positive symptoms	- 3	1.23	-5.104	≤ .001	.91	- 5	13.01	-1.903	.059	
								
	≥ 3	20.30				≥ 5	17.00			

BSI - Positive Symptom Distress Index	- 3	1.15	-3.053	.003	.52	- 5	1.19	-2.151	.033	.37
								
	≥ 3	1.44				≥ 5	1.40			

Other substances use										

Frequency of alcohol use in past month	- 3	3.02	1.266	.208		- 5	2.99	1.030	.305	
								
	≥ 3	2.73				≥ 5	2.75			

Frequency of tobacco use in past month	- 3	2.86	-.875	.383		- 5	2.64	-3.574	.001	.59
								
	≥ 3	3.10				≥ 5	3.54			

Problems with alcohol (RAPI)	- 3	9.93	-2.140	.034	.36	- 5	9.99	-2.342	.021	.41
								
	≥ 3	13.25				≥ 5	13.72			

**Categorical variables**			**Χ**^**2**^	**Sig (2-tailed)**	**Cramer's V**			**Χ**^**2**^	**Sig (2-tailed)**	**Cramer's V**

Cannabis use in past week (yes/no)	- 3		15.54*	≤ .001	.343	- 5		14.43	≤ .001	.331
								
	≥ 3					≥ 5				

Use of other illegal drugs (yes/no)	- 3		2.49*	.114		- 5		11.21	.001	.296
								
	≥ 3					≥ 5				

* Chi-squared value with continuity correction

The table shows significant differences in severity of cannabis use patterns, psychopathological distress and other drugs use between adolescents scoring above and below thresholds (3 points in CPQ-AS, 5 in CAST).

Participants scoring higher than cut-off points in CPQ-A-S and CAST present significantly more problems in RAPI and greater probability of cannabis use in previous week (*p *< .05). CAST cut-off point also indicates differences in lifetime use of other illegal drugs and tobacco use in the month prior to the study (*p *< .05).

According to logistic regression analysis, CPQ-A-S and CAST showed high concurrent predictive validity to predict intensive cannabis use, recent (last week) cannabis use and DSM-IV-TR cannabis abuse and dependence (Table 5).

**Table 5:** Concurrent predictive validity of the CPQ-A-S and the CAST to predict presence of intensive cannabis use in past month (10 times or more), cannabis use in past week, cannabis dependence and cannabis abuse

	B	**S.E**.	Wald	df	**Sig**.	Odds ratio
Intensive cannabis use in past month (10 times or more)						

CPQ-A-S	0.561	0.106	28.230	1	≤ .001	1.753

CAST	0.273	0.056	23.929	1	≤ .001	1.314

Cannabis use in past week						

CPQ-A-S	0.422	0.096	19.276	1	≤ .001	1.524

CAST	0.226	0.058	15.312	1	≤ .001	1.253

Dependence DSM-IV						

CPQ-A-S	0.829	0.134	38.290	1	≤ .001	2.291

CAST	0.512	0.085	36.438	1	≤ .001	1.668

Abuse DSM- IV						

CPQ-A-S	0.472	0.094	25.286	1	≤ .001	1.604

CAST	0.220	0.050	19.061	1	≤ .001	1.246

Logistic regressions and odds ratios.

## Discussion and conclusions

The objectives of the present work were (1) to analyze the structure, reliability and validity of the CAST and CPQ-A-S, (2) to determine their sensitivity and specificity for detecting cannabis dependence, and (3) to compare their capacity for detecting other cannabis-related problems.

Bearing in mind the quality criteria for screening tools, which include brevity, simplicity, sensitivity, specificity and validity, the results show that both instruments are useful for this purpose. Although the canonical correlation between the CAST and the CPQ-A-S is high (0.663), with an associated variance between the two tests of 44%, there are significant differences between them. The CAST is shorter and psychometrically more robust than the CPQ-A-S. The CAST also showed higher specificity in the detection of cannabis dependence. However, no statistically significant differences were found between both tests in their sensitivity and their global discriminative capacity to detect cannabis dependence. The CPQ-A-S appeared as more sensitive to detect psychological distress among cannabis users. Although mean time taken for filling out the questionnaires was not measured, there do not appear to be large differences, and both meet the criteria of brevity and simplicity. Even so, the CAST is a briefer instrument that comprises half as many items as the CPQ-A-S.

### Internal structure of the instruments

Regarding content, the two scales differ in their objectives as well as in their scope with regard to the problems assessed. Most correlations between items from the CPQ-A-S and CAST remain moderate or low, pointing to different areas of assessment and item content between the two questionnaires. Whilst CAST focuses more on detection of cannabis use disorders, CPQ-A-S explores a broader spectrum of problems commonly associated with the use of this drug. Additionally, CPQ-A-S and CAST differ in the assessment period they use and their response format. The CAST requests information on a 12-month period, whereas the CPQ-A-S obtains data only on the last 3 months. Moreover, the CAST uses a Likert-type response system (from 0 to 4), as against the dichotomous nature of the CPQ-A-S response format. These differences might be contributing to the CAST obtaining greater values of internal consistency compared to the CPQ-A-S.

In line with the findings of previous works on the CPQ-A-S [11], this instrument shows an essentially one-dimensional structure, as is the case of the CAST. The Cronbach's alpha coefficients of the CPQ-A-S obtained in this study and in the original validation are very similar [11], at 0.70 and 0.73, respectively. Both the CAST and the CPQ-A-S have good internal consistency, with alpha coefficients of over 0.70. The CAST, though, has a reliability index higher than that of the CPQ-A-S, attaining a value of 0.84, similar to that of the original study [14], in spite of its being shorter. It should be taken into account that the dichotomous response format used in the CPQ-A-S tends to generate lower correlations than the Likert-type format employed in the CAST [31]. Likewise, given that these instruments address different areas of the cannabis user's life that are not necessarily related, modest internal consistency values are to be expected.

### Screening of cannabis dependence

Both instruments show high sensitivity and specificity for detecting cannabis users with dependence according to the DSM-IV-TR criteria, the differences between the two tests being slight. Both the CPQ-A-S and the CAST show, with their respective cut-off points, a sensitivity of 83%, leaving out 17% of positive cases, without statistically significant differences. As regards specificity, the CAST incorrectly classifies as positive just 13%, whilst this figure rises to 22.5% in the case of the CPQ-A-S. According to our results, the CAST is significantly more specific that the CPQ-A-S. Although in total, the CPQ-A-S correctly classifies 79.2% of cases, versus 85.4% for the CAST, no significant differences emerge regarding global discriminative capacity. According to our results, differences in areas under the ROC Curve are not statistically significant. The CAST is therefore a briefer and more reliable instrument than the CPQ-A-S and it seems to be more specific to detect cannabis dependence, but further studies should be undertaken to confirm significant differences in their discriminative capacity.

### Detection of other cannabis-related problems

As far as concurrent predictive validity is concerned, both instruments are useful for detecting a more serious pattern of cannabis use. Moreover, each of the instruments detects a different profile of problematic cannabis user, pointing to different clinical needs. Young people scoring above the cut-off points are more likely to have used cannabis recently, to have begun using it earlier, to have used it for longer and to be more concerned about its effects and in more areas of their life. They are also likely to have problems associated with excessive drinking, as assessed by the RAPI. Furthermore, CAST scores are also associated with tobacco use in the past month and with the use of other illegal drugs. On the other hand, the CPQ-A-S presents an effect size markedly larger than that of the CAST in the detection of consumer's global concern about the effects of cannabis, highlighting the clinical relevance of the problems assessed.

The CPQ-A-S also appears to be more sensitive in the detection of psychopathological distress. The cut-off point set for this instrument detects statistically significant differences in all the dimensions assessed by the BSI, whilst the CAST detects no differences in psychopathological distress according to 8 of the 13 scales. Moreover, the effect size is larger in all cases for the CPQ-A-S. It seems that the problems assessed by the CPQ-A-S derive not only from use of the drug, but also from the interaction between its use and the user. The methodology employed does not permit us to determine whether the higher scores in psychopathological symptoms are previous to cannabis use or subsequent to it. Even so, the CPQ-A-S emerges as a more appropriate tool for detecting those young cannabis users who, apart from consuming more, present higher levels of psychopathological distress.

Finally, the CPQ-A-S presents in all cases a predictive capacity higher than that of the CAST with regard to recent use, intensive use and the presence of cannabis abuse and dependence according to the DSM IV criteria. The differences are not large in any of the cases.

## Conclusions

In conclusion, both the CAST and the CPQ-A-S are reliable and valid screening instruments for problematic cannabis use in young people. The CAST is shorter and has slightly higher internal consistency as well as higher specificity to detect cannabis dependence. The CPQ-A-S, on the other hand, appears to be better at detecting profiles of cannabis users with psychopathological symptoms associated with frequent use of the drug. Perhaps for this reason it is a better detector of users with more concern about their cannabis use.

As shown by the results, cannabis users scoring above established thresholds present not only more severe patterns of cannabis use, with the associated health risks, but also greater psychological distress. The use of these tools is essential for a quick and early screening that permits referral of adolescents who might be at risk and in need of professional intervention. All the more so when adolescents are often reluctant to seek help on their own [7]. Results of the present study offer professionals guidance to detect adolescents in need of further assessment, and eventually, a referral to treatment programs.

## Limitations

These results must be interpreted taking into account some of the limitations of our study. The first of these limitations is the lack of indicators external to the assessment that permit the validation of the screening results. In this regard, it would be useful to obtain clinical judgements by professionals about the presence of cannabis use disorders. The cost of the procedure made it impossible to obtain this type of data, even though it is customary to obtain it through self-report in research studies [23]. Nor was it possible to carry out a test-retest study for determining the temporal stability of the scores, due to the cross-sectional design of the research. Additionally, results of the study are limited to those adolescents with any cannabis use in the previous month. Further investigations could compare screening properties of the instruments among a sample of last-year cannabis users and also using different gold standards, such as presence of cannabis abuse. Given the differences found between the detection capacity of the two instruments, it may be that the use of a larger sample would permit the identification of subgroups of cannabis users with different patterns of use and problems, so as to determine with more accuracy the profile identified by each of the instruments. Using a larger sample size would also help overcome some limitations of statistical power in the present study, and therefore increase reliability of results.

## Competing interests

The authors declare that they have no competing interests.

## Authors' contributions

SFA participated in the design of the study, collected data and drafted the manuscript. JRFH had the original idea for the study and its design, and helped draft the manuscript. JMF participated in the design and performed the statistical analysis. RSV participated in the design of the study and its coordination. GGF helped with the data collection and the drafting of the manuscript. All authors significantly contributed to the project and eventually read and approved the manuscript.
